# Global research trends in music therapy for surgery: a bibliometric analysis (2009–2023)

**DOI:** 10.1186/s13741-025-00496-x

**Published:** 2025-01-30

**Authors:** Xiaoping Xu, Zhenglan Zhong, Yong Yi

**Affiliations:** 1 Department of Neurosurgery, the Second People’s Hospital of Yibin, Yibin, 644000 Sichuan Province China; 2 Department of Sterilization and Supply Center, the Second People’s Hospital of Yibin, Yibin, 644000 Sichuan Province China

**Keywords:** Music therapy, Surgery, Bibliometric analysis, VOSviewer, CiteSpace

## Abstract

Music therapy, known for its profound impact on human emotions and physiology, has gained increasing attention for its applications in medical settings, particularly in surgery. This study conducted a bibliometric analysis of publications on the application of music therapy in surgery from 2009 to 2023, utilizing the Web of Science Core Collection (WoSCC) as the primary database. A total of 479 publications were analyzed using VOSviewer, CiteSpace, Microsoft Excel, and online bibliometric tools. Findings indicate a steady increase in annual publications since 2009, peaking in 2021. The USA leads global research efforts with 31.7% of publications, followed by China (17.7%) and Italy (10%). Harvard University was identified as the top contributing institution, while the *Journal of Perianesthesia Nursing* was the primary publishing journal, and the *Cochrane Database of Systematic Reviews* was the highest co-cited journal. Cao Hua contributed the most publications, and Nilsson U was identified as the most co-cited author (*n* = 131). Keyword analysis highlighted anxiety, therapy, music therapy, and pain as primary research trends. This study provides valuable insights into the evolving landscape of music therapy research in surgical contexts. Future efforts should focus on expanding interdisciplinary collaborations, exploring advanced technologies for personalized interventions, and investigating optimal implementation strategies to enhance the integration of music therapy into surgical practice.

## Introduction

Surgery represents a significant and often daunting experience for patients, frequently accompanied by heightened levels of anxiety, fear, and discomfort. Studies indicate that approximately 75% of pre-operative surgical patients experience considerable anxiety (Oliveira et al. 2014; Robleda et al. 2014). Elevated anxiety levels, coupled with pain, can lead to negative physiological effects, adversely impacting surgical outcomes and prolonging recovery times. In response to the growing emphasis on holistic patient care, healthcare professionals have increasingly adopted adjunctive therapies to address these challenges.

The integration of music within surgical settings, first explored in the early twentieth century, emerged as a pioneering effort to reduce patient stress and improve clinical outcomes. Early work by Evan Kane highlighted music’s potential to create a soothing environment in the operating room (Kane 1914). Subsequent research has provided robust evidence supporting the efficacy of music therapy in surgical care, demonstrating its ability to reduce preoperative anxiety, alleviate pain, and enhance recovery processes (Millett and Gooding 2018; Keenan and Keithley 2015; Hartling et al. 2013; Mackintosh et al. 2018; Zhou et al. 2022). The therapeutic effects of music are underpinned by mechanisms such as modulation of autonomic nervous system activity, regulation of stress hormone secretion, and engagement of reward pathways in the brain (Salimpoor et al. 2011; Smith et al. 2021).

In recent years, music therapy has gained increasing attention across diverse medical contexts as a non-invasive, safe, and cost-effective intervention with no known specific side effects. Compared to pharmacological methods, music interventions offer a unique approach to enhancing patient well-being. Empirical studies have underscored its significant positive effects on various surgical procedures (Jiang et al. 2024; Araujo-Duran et al. 2024; Aksu 2023; Jacquier et al. 2022; Bertacco et al. 2022; Guerrier et al. 2021). A comprehensive meta-analysis of 73 randomized controlled trials, encompassing a total of 6902 patients, demonstrated that music therapy was associated with moderate reductions in patient-reported postoperative pain and anxiety, decreased use of analgesics, and improved patient satisfaction. Importantly, none of the included studies reported any adverse side effects, underscoring the safety profile of this intervention (Hole et al. 2015).

Despite the growing body of research on music therapy, the application of bibliometric analysis to systematically assess trends in this domain remains limited. Bibliometric analysis provides a powerful tool to examine publication trends, citation patterns, and thematic content, offering insights into the evolution of research, identifying key contributors, and evaluating the broader impact of music therapy on surgical outcomes. Notably, while bibliometric studies on music therapy have been conducted, a focused analysis of its application in surgery is lacking. This study aims to identify key contribFTutors, analyze current research trends, and explore future directions in the application of music therapy within surgical contexts through bibliometric analysis.

## Materials and methods

### Data source and search strategy

The Web of Science Core Collection (WoSCC) was selected as the primary database for this study due to its comprehensive and systematic coverage of high-quality academic journals. Compared to other databases such as Scopus, PubMed, and Medline, WoSCC is frequently utilized in bibliometric studies due to its reliability and extensive citation-tracking capabilities(Yeung 2019; Birkle et al. 2020; Gusenbauer 2024; Daugherty et al. 2022). Previous studies have underscored the effectiveness of employing bibliometric analysis using the WoSCC database (Wang and Maniruzzaman 2022; Abumalloh et al. 2024). In this study, we leveraged this resource to retrieve pertinent literature published between January 1, 2009, and December 31, 2023. Our search criteria were defined as follows: TS = (“Music therapy”) AND TS = (“Surgery”).

### Inclusion and exclusion criteria

**Fig. 1:**
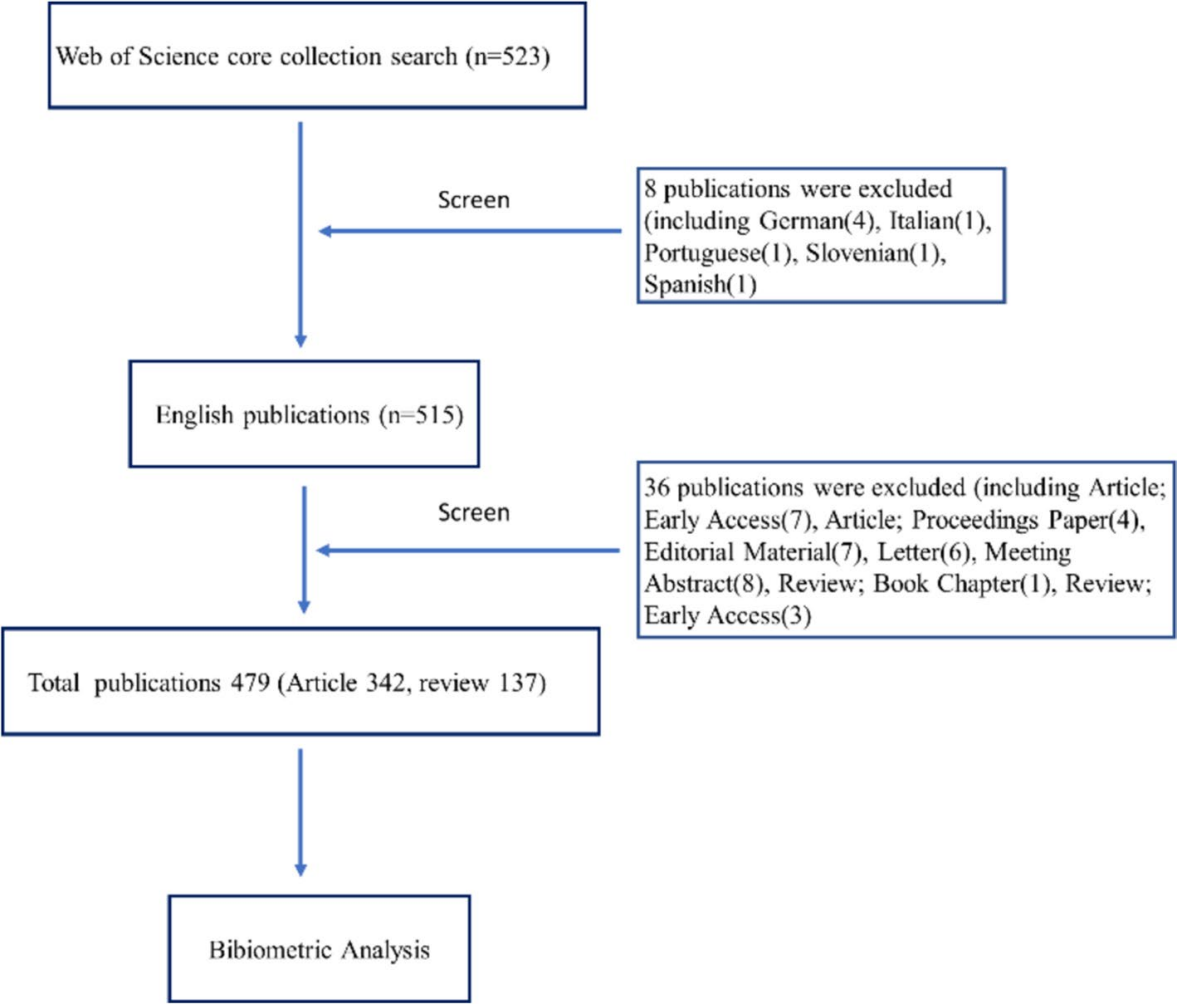
The study flow diagram for screening

Following the screening of titles and abstracts, we exclusively focused on articles and reviews, excluding other irrelevant literature such as meeting abstracts, letters, editorial material, book chapters, and non-English papers. Additionally, we eliminated duplicated articles. Prior to analysis, two researchers independently screened the data, resolving any discrepancies through discussion and with the intervention of a third reviewer when necessary. The inclusion criteria flowchart is depicted in Fig. 1. Ultimately, a total of 479 records were retrieved for the bibliometric analysis.

### Bibliometric analysis

The data were analyzed using a combination of VOSviewer, CiteSpace, Microsoft Excel 2021, and an online bibliometric tool (https://bibliometric.com/).

VOSviewer (version 1.6.20) was employed to examine relationships among authors, countries, institutions, journals, references, and keywords. This software is widely recognized for its ability to extract key insights from extensive publication datasets (Eck and Waltman 2009).

To uncover the interconnections between different journals within our field of study, we utilized CiteSpace (version: 6.3.1) to generate a dual-map overlay of journals. Additionally, CiteSpace was utilized to analyze reference patterns, leveraging its Citation Bursts feature to identify significant shifts or bursts in citation activity for specific references over time.

Quantitative analysis of publication metrics was conducted using Microsoft Office Excel 2021.

For visualizing trend topics, we utilized the online bibliometric website (https://bibliometric.com/).

## Results

### Quantitative analysis of publication

**Fig. 2:**
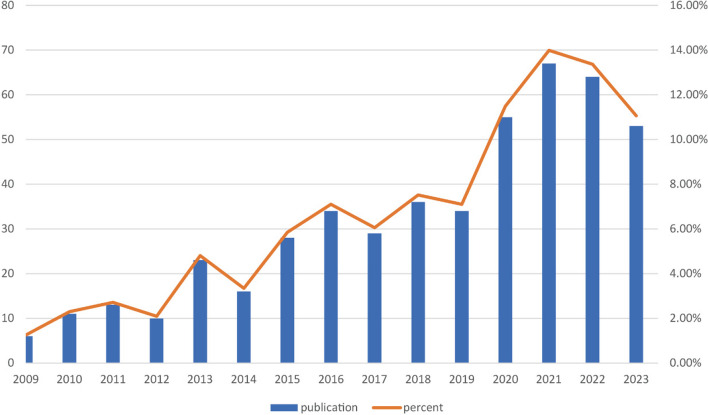
Annual output of research on the application of music therapy in surgery

In Fig. 1, spanning the period from 2009 to 2023, a total of 523 studies were identified within the WoS Core Collection. Following a comprehensive review of full articles, 8 non-English publications and 36 publications in other formats were excluded. Ultimately, a corpus of 479 studies on the application of music therapy in surgical settings was compiled, comprising 342 articles and 137 reviews. The yearly distribution of publications is depicted in Fig. 2, revealing a consistent upward trajectory from 2009 onwards, peaking in 2021 before experiencing a modest decline between 2022 and 2023. This trend underscores the sustained and enduring attention accorded to this subject matter over the years. Notably, the highest number of published articles (*n* = 67) was recorded in 2021. The decline in publications after 2021 may be attributed to several factors, including the impact of the COVID-19 pandemic, which shifted research priorities and resources, and possible delays in the peer-review and publication process. Additionally, changes in funding allocation and a natural shift in research focus toward emerging technologies, such as virtual reality or artificial intelligence, may have contributed to this trend. These factors warrant further investigation to better understand their influence on research activity in this field.

### Country and institutional analysis

To explore the prominent contributors in this field, an analysis of the distribution of publications by countries and institutions was conducted. Table 1 reveals that among 61 countries, the USA holds the highest number of publications (*n* = 152, 31.7%), followed by China (85, 17.7%) and Italy (48, 10%). Together, the publications from these top three countries represent over half of the total (*n* = 285, 59.5%), while the remaining countries contribute less than 10% of the total publications. These findings underscore the leading role of the USA in music therapy research. These remarkable advancements can be largely attributed to the strong institutional support provided by both the USA and China, including favorable policies and substantial financial funding. Such support has enabled extensive and in-depth research in this field. Additionally, other contributing factors may include the increasing emphasis on interdisciplinary collaboration and the growing recognition of music therapy’s potential to improve surgical outcomes, which collectively drive innovation and progress in this area.

**Table 1:** Top 10 countries and institutions in research on the application of music therapy in surgery

Rank	Country	Counts	Institution	Counts
1	USA	152(31.7%)	Harvard University	16(3.3%)
2	Peoples R China	85(17.7%)	Fujian Medical University	11(2.3%)
3	Italy	48(10%)	Harvard Medical School	10(2.1%)
4	Germany	32(6.7%)	University System of Ohio	10(2.1%)
5	Turkey	32(6.7%)	Taipei Medical University	10(2.1%)
6	Iran	26(5.4%)	University of Michigan	10(2.1%)
7	England	24(5%)	University of Michigan System	10(2.1%)
8	Taiwan	21(4.4%)	Sichuan University	8(1.7%)
9	Canada	18(3.8%)	University of California System	8(1.7%)
10	Netherlands	16(3.3%)	University of London	8(1.7%)

**Fig. 3:**
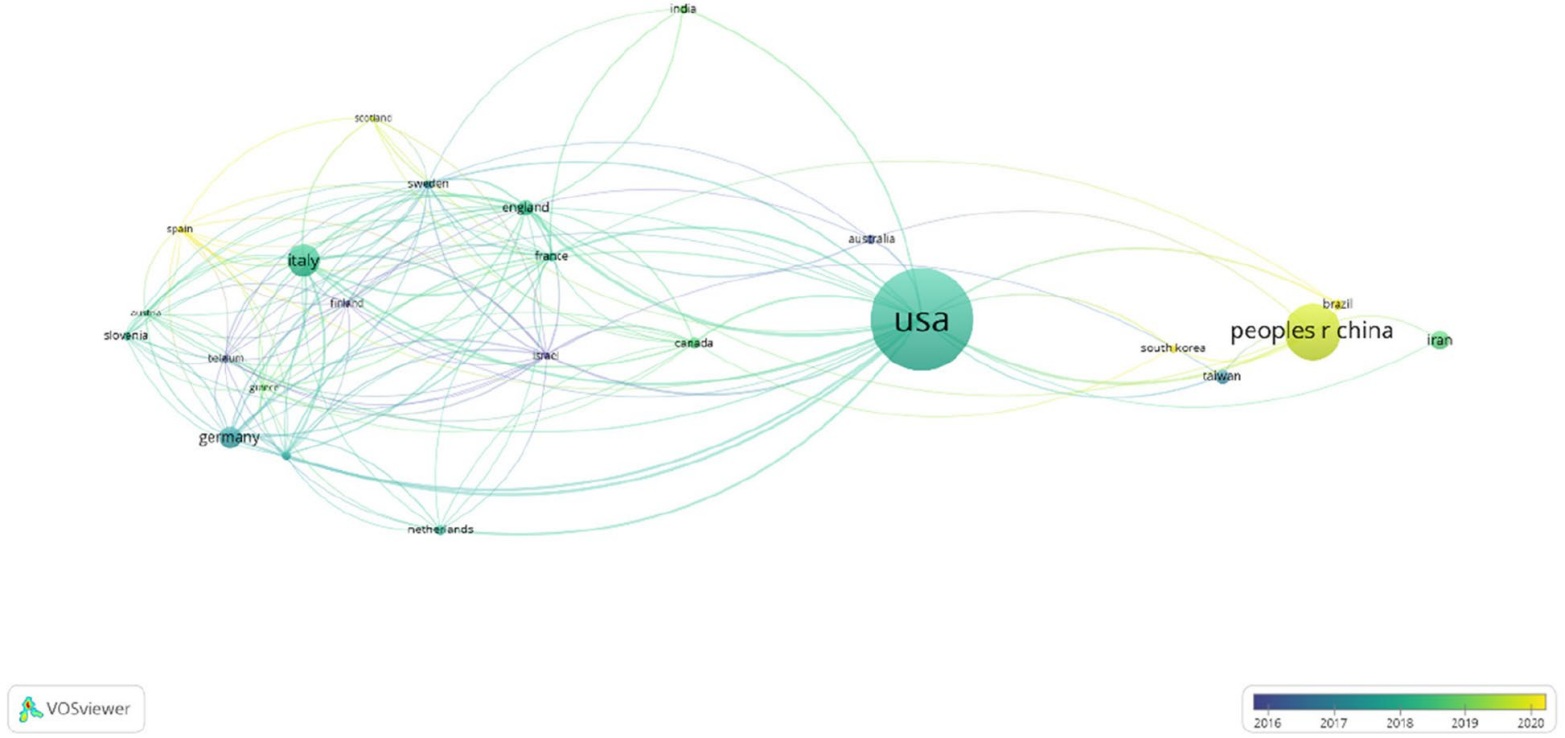
A network map showing countries involved in research on the application of music therapy in surgery

**Fig. 4:**
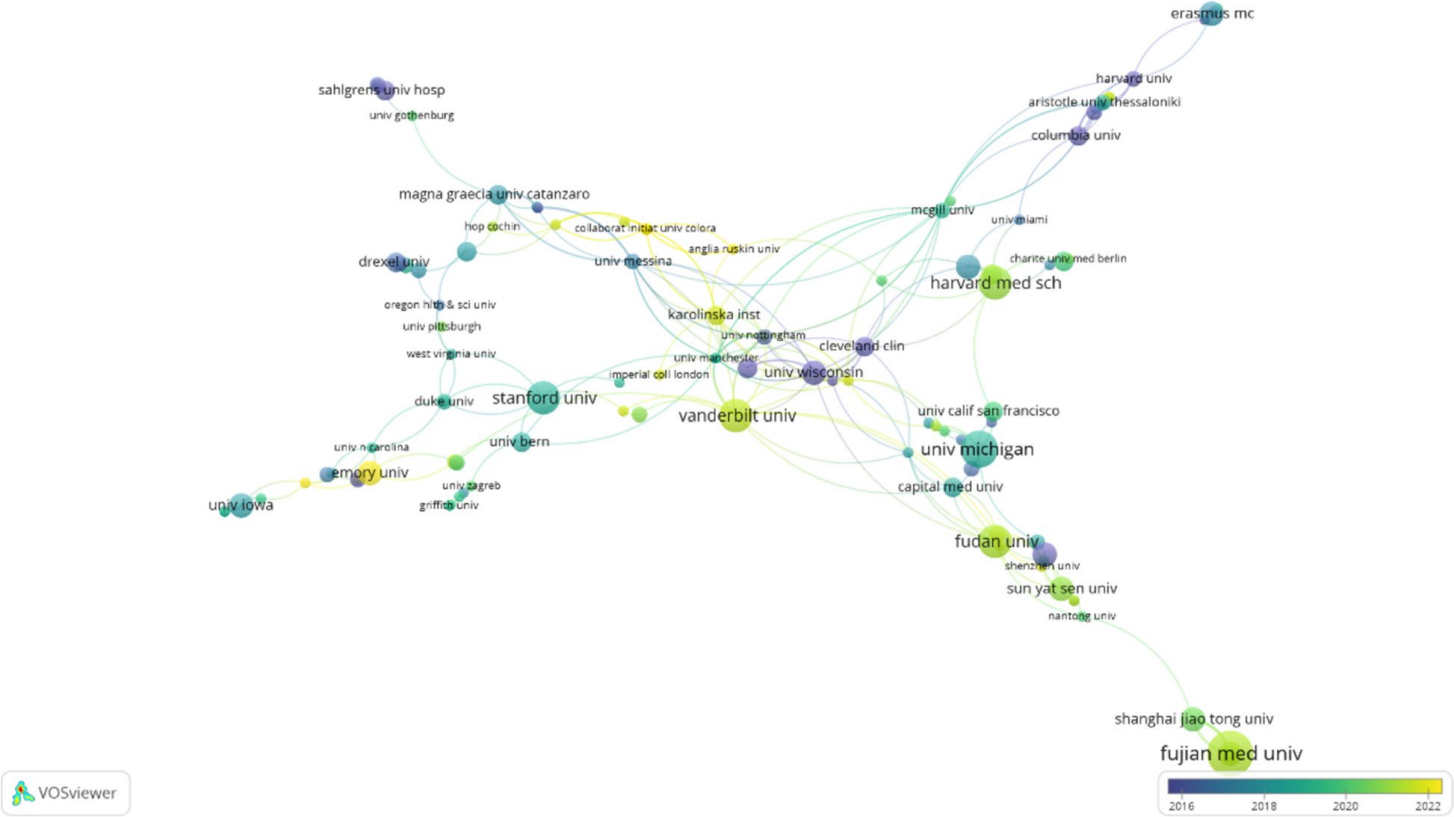
A network map showing institutions involved in research on the application of music therapy in surgery

Collaborative networks among various countries were visualized using VOSviewer. In the visualization map, each color corresponds to a distinct cluster, the size of the nodes represents the volume of publications, and the thickness of the links signifies the strength of collaborative interactions. As illustrated in Fig. 3, the USA engages in active collaboration with numerous countries, including England, Germany, Italy, China, and the Netherlands. Further analysis revealed that these articles were contributed by a total of 948 institutions. Figure 4 visually depicts the collaborative network, illustrating the quantity and interrelationships of publications from each institution.

### Journals and co-cited journals

**Fig. 5:**
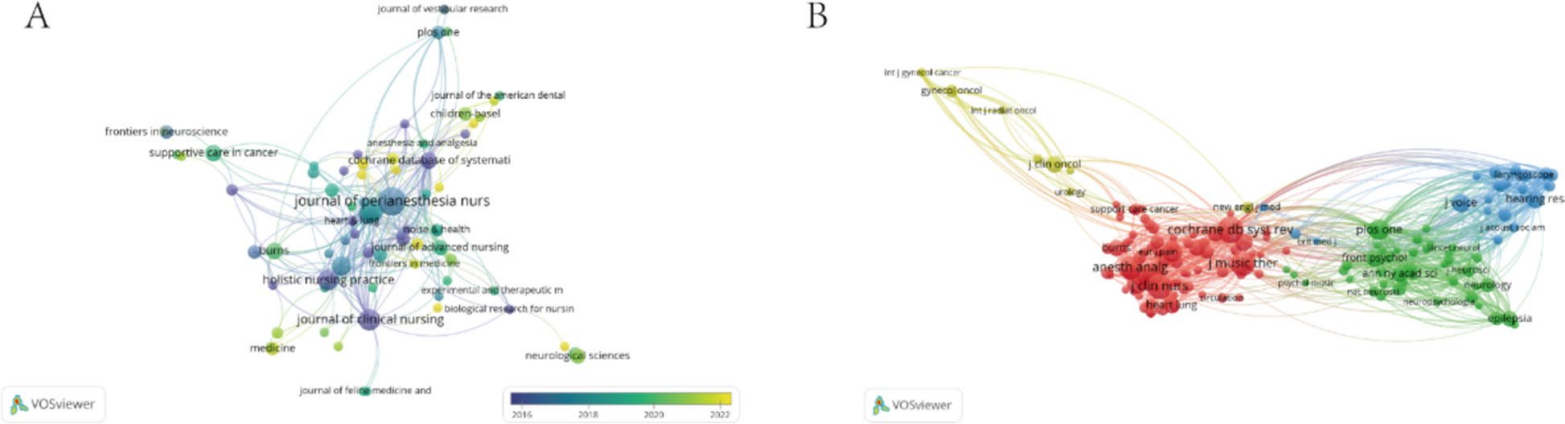
Visualization of journals (**A**) and co-cited journals (**B**) in research on the application of music therapy in surgery

A total of 479 studies included in our bibliometric analysis were distributed across 283 journals. Table 2 presents the top 10 journals and co-cited journals in the research field of music therapy applied in surgery. The most prolific journal was *Journal of Perianesthesia Nursing* (*n* = 17, 3.5%), followed by *Complementary Therapies in Clinical Practice* (*n* = 11, 2.3%) and *Journal of Clinical Nursing* (*n* = 10, 2.1%). Analysis of co-cited journals using VOSviewer revealed that the *Cochrane Database of Systematic Reviews* had the highest co-citation count (309), followed by *the Journal of Clinical Nursing* (286), and the *Journal of Music Theory* (272). Figure 5 displays the network of journals (Fig. 5A) and co-cited journals (Fig. 5B). The thickness of the lines between the two items indicates the strength with which they cited each other. The significant co-citation count signifies the journals with the most significant academic influence in the field, with the *Cochrane Database of Systematic Reviews* holding key positions in this research domain.

**Table 2:** Top 10 journals and co-cited journals for research of music therapy applied in surgery

Rank	Journal	Count	Co-Cited Journal	Co-citation
1	Journal of Perianesthesia Nursing	17(3.5%)	Cochrane Database Of Systematic Reviews	309
2	Complementary Therapies In Clinical Practice	11(2.3%)	Journal Of Clinical Nursing	286
3	Journal of Clinical Nursing	10(2.1%)	Journal Of Music Theory	272
4	Complementary Therapies In Medicine	9(1.9%)	Anesthesia & Analgesia	247
5	Holistic Nursing Practice	9(1.9%)	Journal Of Advanced Nursing	226
6	Pain Management Nursing	8(1.7%)	Plos One	188
7	Cochrane Database of Systematic Reviews	6(1.3%)	Pain	184
8	Burns	6(1.3%)	Pain Management Nursing	183
9	Journal of Voice	6(1.3%)	Aorn Journal	181
10	Supportive Care In Cancer	6(1.3%)	Journal Of Perianesthesia Nursing	153

**Fig. 6:**
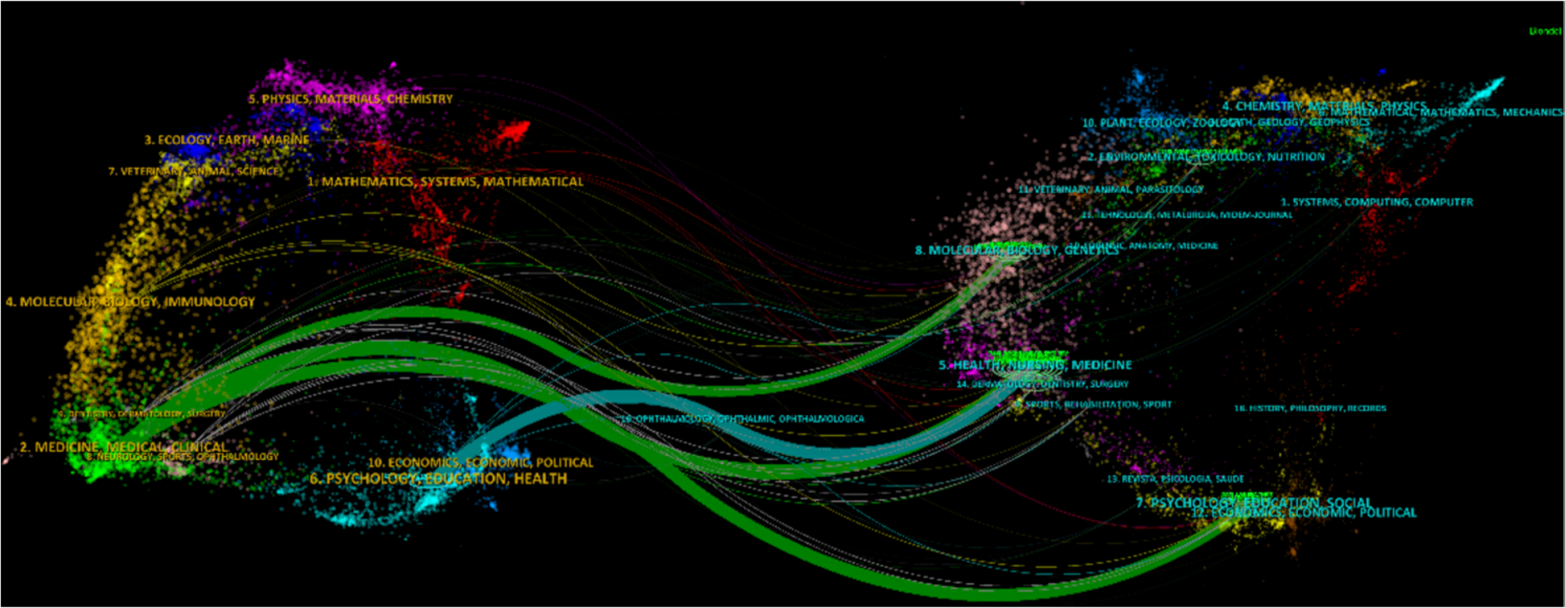
Dual-map overlay of journals in research on the application of music therapy in surgery

CiteSpace software was utilized to visually depict the dual-map overlaps of the journals and co-cited journals. Each color corresponds to a specific discipline associated with a journal, while the curves illustrate the citation pathways. The thickness of each curve indicates the frequency of cross-disciplinary citations, with thicker curves signifying stronger connections and closer interactions between the respective fields. As illustrated in Fig. 6, four main citation trajectories were identified, journals in medicine/medical/clinical are mainly cited by Health/Nursing/Medicine, Psychology/Education/Social and Psychology/Education/Social and Molecular/Biology/Genetics fields. Additionally, journals in Psychology/Education/Health are mainly influenced by journals in Health/Nursing/Medicine fields.

### Authors and co-cited authors

**Fig. 7:**
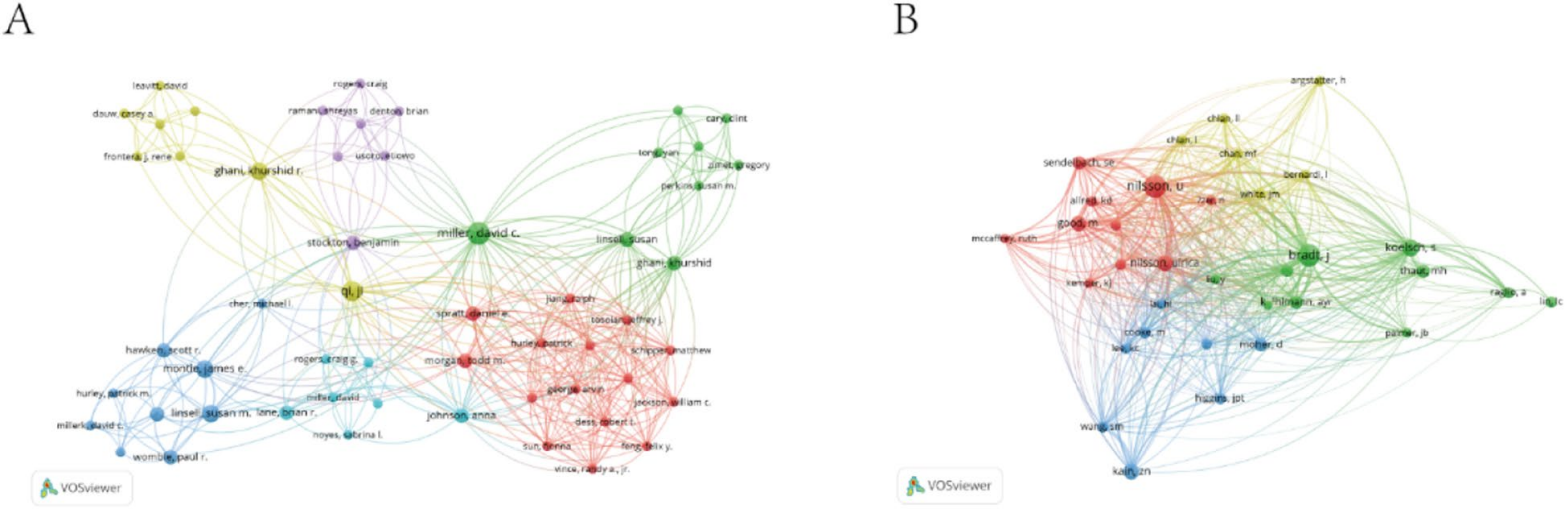
Visualization of authors (**A**) and co-cited authors (**B**) in research on the application of music therapy in surgery

A total of 2433 authors contributed to the 479 selected publications. Author Cao Hua published the highest number of studies (*n* = 8), followed by Chen Qiang (*n* = 8), Johannes Jeekel (*n* = 6), and David C. Miller (*n* = 5). There were a total of 14,184 co-cited authors, Among them, Nilsson U (*n* = 131) and Bradt J (*n* = 125) emerged as the top two co-cited authors. A cooperative network based on authors and co-cited authors was constructed using VOSviewer (Fig. 7). The varying colors of the circles represent distinct clusters of authors or groups of co-cited authors. The thickness of the lines reflects the strength of the relationships, with thicker lines indicating stronger connections. The network map illustrates authors who have demonstrated high levels of cooperation in their productivity, forming distinct clusters.

### Co-cited references

**Fig. 8:**
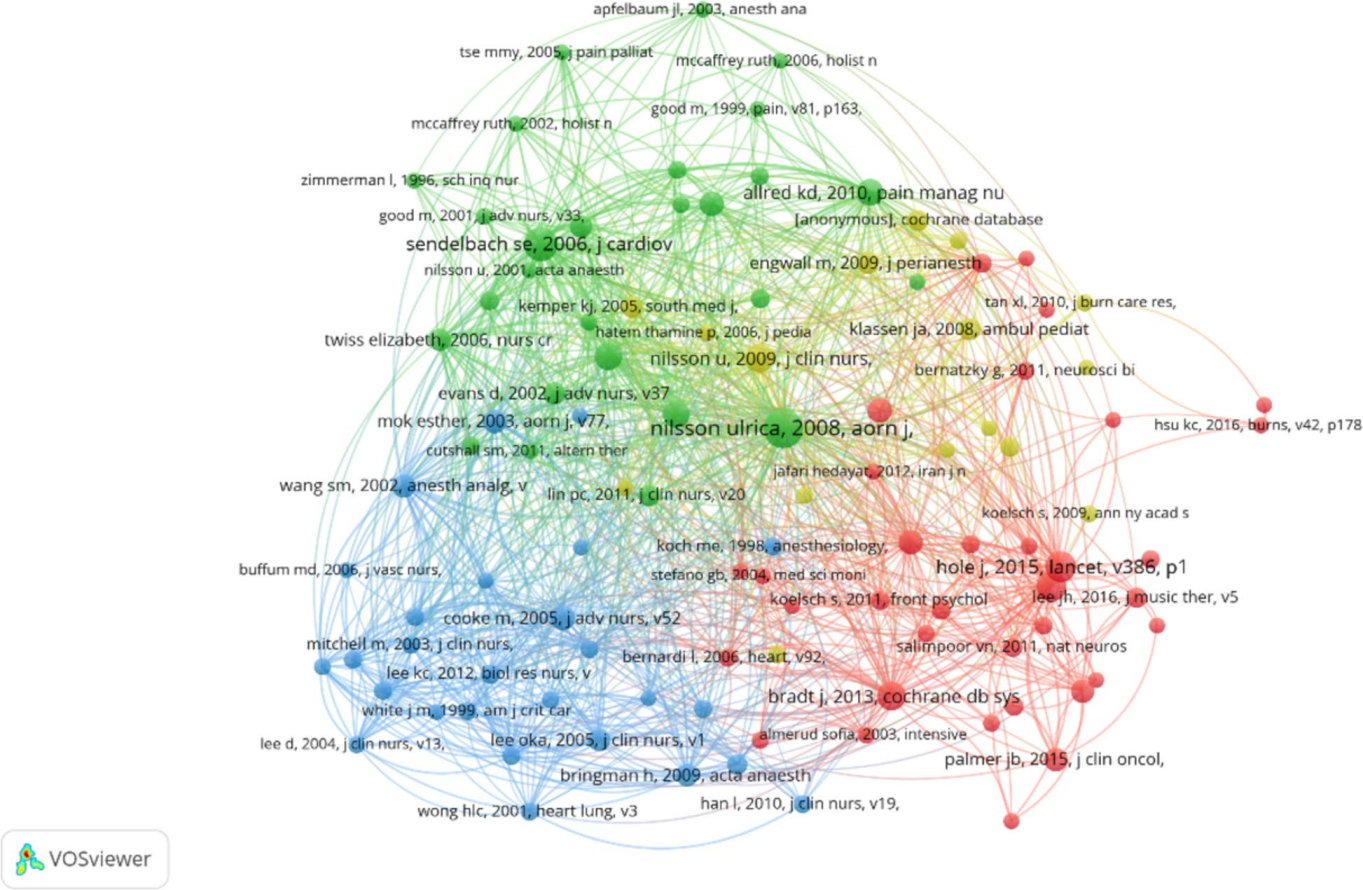
Visualization of co-cited references in research on the application of music therapy in surgery

Over the past 16 years, there have been 17,738 co-cited references related to the application of music therapy in surgery. Table 3 presents the top 10 co-cited references in this area, with seven references being co-cited more than 30 times. Figure 8 illustrates the co-citation network map based on selected references co-cited 10 times or more, resulting in 4 clusters (105 items). “nilsson Ulrica, 2008, AORN J” demonstrates active co-cited relationships with “sendelbach se, 2006, J CARDIOV”, and “allred kd, 2010, PAIN MANAG NURS” etc.

**Table 3:** Top 10 co-cited references on the application of music therapy in surgery

Rank	Co-cited reference	Citations
1	nilsson ulrica, 2008, aorn j, v87, p780. https://doi.org/10.1016/j.aorn.2007.09.013	62
2	sendelbach se, 2006, j cardiovasc nurs, v21, p194. 10.1097/00005082-200605000-00007	46
3	hole j, 2015, lancet, v386, p1659 doi:10.1016/s0140-6736(15)60169-6	39
4	kühlmann ayr, 2018, brit j surg, v105, p773. https://doi.org/10.1002/bjs.10853	35
5	bradt j, 2013, cochrane db syst rev https://doi.org/10.1002/14651858.cd006908.pub2	34
6	nilsson u, 2009, j clin nurs, v18, p2153.https://doi.org/10.1111/j.1365-2702.2008.02718.x	34
7	leardi s, 2007, brit j surg, v94, p943. https://doi.org/10.1002/bjs.5914	31
8	allred kd, 2010, pain manag nurs, v11, p15. 10.1016/j.pmn.2008.12.002	29
9	nilsson u, 2009, heart lung, v38, p201.10.1016/j.hrtlng.2008.07.008	28
10	Özer n, 2013, pain manag nurs, v14, p20.https://doi.org/10.1016/j.pmn.2010.05.002	25

### Reference with citation burst

**Fig. 9:**
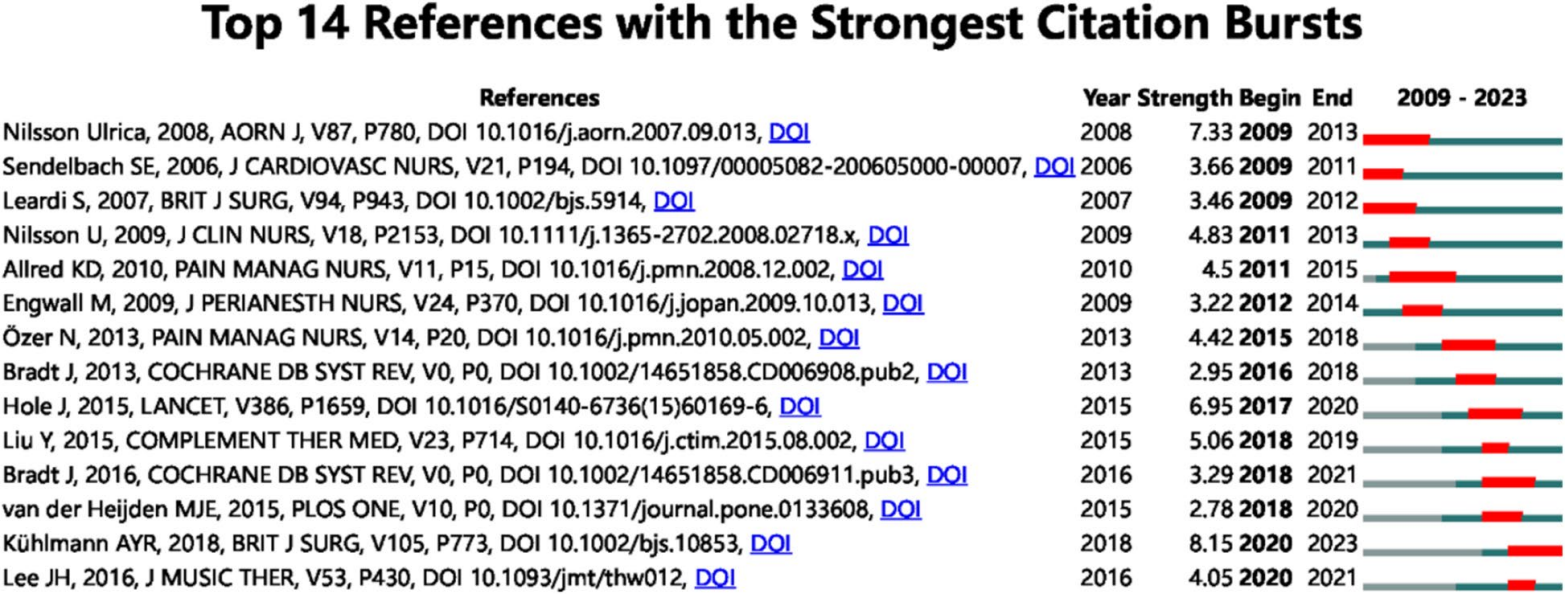
Top 14 references with strong citation bursts. A red bar indicates high citations in that year

In Fig. 9, by using CiteSpace, we present 14 references that exhibited the most significant citation bursts in our study. The initial occurrence of a citation burst was observed as early as 2009, the burst strength of these references ranged from 2.78 to 8.15, with endurance strength from 2 to 5 years. Notably, The reference with the strongest citation burst (strength = 8.15) was titled “Meta-analysis evaluating music interventions for anxiety and pain in surgery” published in the British Journal of Surgery by A Y R Kühlmann et al. with citation bursts spanning from 2009 to 2013. Table 4 presents a concise overview of the principal research themes delineated across the 14 references, arranged in accordance with the sequence depicted in Fig. 9 of the literature.

**Table 4:** The main research contents of the 14 references with strong citation bursts

Rank	Strength	Main research content
1	8.15	Meta-analysis evaluating music interventions for anxiety and pain in surgery
2	7.33	The Anxiety- and Pain-Reducing Effects of Music Interventions: A Systematic Review
3	6.95	Music as an aid for postoperative recovery in adults: a systematic review and meta-analysis
4	5.06	Effects of music therapy on pain, anxiety, and vital signs in patients after thoracic surgery
5	4.83	Soothing music can increase oxytocin levels during bed rest after open-heart surgery: a randomised control trial
6	4.5	The Effect of Music on Postoperative Pain and Anxiety
7	4.42	Effect of Music on Postoperative Pain and Physiologic Parameters of Patients after Open Heart Surgery
8	4.05	The Effects of Music on Pain: A Meta-Analysis
9	3.66	Effects of Music Therapy on Physiological and Psychological Outcomes for Patients Undergoing Cardiac Surgery
10	3.46	Randomized clinical trial examining the effect of music therapy in stress response to day surgery
11	3.29	Music interventions for improving psychological and physical outcomes in cancer patients
12	3.22	Music as a Nursing Intervention for Postoperative Pain: A Systematic Review
13	2.95	Music interventions for preoperative anxiety
14	2.78	The Effects of Perioperative Music Interventions in Pediatric Surgery: A Systematic Review and Meta-Analysis of Randomized Controlled Trials

Hotspots and frontiers

**Fig. 10:**
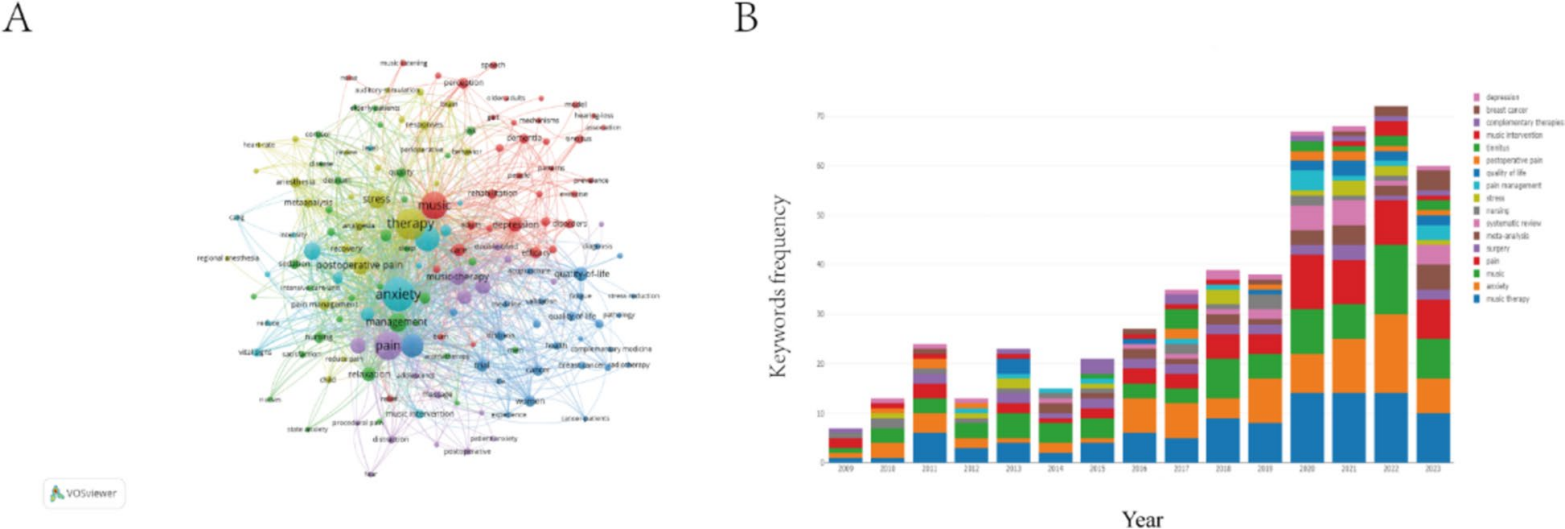
Keyword cluster analysis (**A**) and trend topic analysis (**B**)

The results of the keywords analysis revealed the research hotspots within the field. Table 5 presents the top 10 keywords with the strongest citation bursts from 2009 to 2023. Notably, “anxiety” received the most sustained attention (counts = 171), followed closely by “therapy” (counts = 149), “music therapy” (counts = 147), “pain” (counts = 127), and “music” (counts = 116). Keywords with counts lower than 100 were observed for the remaining terms. Furthermore, we filtered all keywords with a minimum occurrence of 10 and conducted cluster analysis using VOSviewer. This process yielded five major clusters, indicating five distinct research fields (Fig. 10A).

**Table 5:** Top 10 keywords in research on application of music therapy in surgery

Rank	Keywords	Counts	Rank	Keywords	Counts
1	Anxiety	171	6	Surgery	85
2	Therapy	149	7	Management	57
3	Music therapy	147	8	Stress	51
4	Pain	127	9	Postoperative pain	49
5	Music	116	10	Intervention	46

Additionally, we utilized the online bibliometrics website (https://bibliometric.com/) to further explore the trending topics. The results indicated that from 2020 to 2023, research during this period predominantly focused on music therapy, indicating a potential future research direction (Fig. 10B).

## Discussion

In the present study, we observed a sustained increase in annual publications on music therapy since 2009, peaking in 2021. This surge highlights the growing academic and clinical interest in music therapy, particularly in surgical contexts. Notably, the USA and China emerged as primary contributors, reflecting robust research funding, infrastructure, and academic influence. Harvard University, a premier research institution, plays a pivotal role in advancing this field, benefiting from interdisciplinary collaboration and a legacy of innovation. Additionally, the *Journal of Perianesthesia Nursing* and the *Cochrane Database of Systematic Reviews* were identified as influential venues, indicating a focus on high-impact and evidence-based studies. Cao Hua, as the most prolific author, and Nilsson U as the most frequently co-cited researcher, have emerged as key contributors, highlighting their significant impact and lasting influence in advancing this field. Keyword analysis revealed that anxiety, music therapy, and pain were the primary research trends in recent years, reflecting the evolving priorities and therapeutic potential of this field.

To the best of our knowledge, this is the inaugural bibliometric analysis focusing on the utilization of music therapy within surgical contexts. Previous bibliometric analyses have primarily explored broader aspects of music therapy. Lun T et al. highlighted anxiety management as a key focus in music therapy, noting its growing recognition for improving psychological well-being in clinical settings (Lun et al. 2024). Zhi L et al. analyzed global trends in music therapy research, emphasizing the field’s rapid growth, increasing diversity of approaches, and the role of multidisciplinary collaborations in advancing the discipline (Zhi et al. 2013). Similarly, Kailimi Li et al. highlighted the dominance of the USA in music therapy publications and its significant institutional influence (Li et al. 2021). Shao Yin et al. analyzed music therapy for dementia patients, emphasizing priorities like quality of life enhancement, personalized therapy approaches, and caregiver well-being (Yin et al. 2022). Additionally, bibliometric inquiries into non-pharmacological interventions, such as art therapy (Liu et al. 2021) and rhythmic auditory stimulation (Zhang et al. 2022), have highlighted the expanding interest in employing complementary therapies across diverse clinical settings. Collectively, these studies have hinted at the burgeoning interest in employing music therapy across diverse clinical settings, foreshadowing its emergence as a focal point of future research endeavors. In contrast to these previous analyses, our study provides a novel perspective by concentrating exclusively on the integration of music therapy within surgical practice—a domain where its potential benefits remain underexplored. By concentrating on this specific domain, we provide a detailed analysis of current research trends, key contributors, and thematic focuses, offering a nuanced understanding of how music therapy has been integrated into surgical care. Moreover, it highlights actionable insights that could inform future multidisciplinary collaborations, ultimately driving innovation and improving patient-centered outcomes in perioperative settings.

Consistent with earlier bibliometric analyses, our findings suggest that developed nations will continue to dominate this field due to their advanced resources and institutional strengths. However, a noteworthy trend is the increasing interest from developing countries, likely driven by advancements in technology and greater access to global research networks. Li et al. noted a diversification of contributors in music therapy research, pointing to an expansion of the global research ecosystem (Li et al. 2021). Future studies could explore how region-specific challenges and cultural contexts influence research outputs and priorities in these areas.

This study offers a comprehensive lens to examine recent advancements and trends in music therapy applications in surgical settings, serving as a valuable resource for researchers. Moving forward, greater emphasis on global collaboration, interdisciplinary research, and personalized therapeutic approaches will be essential in unlocking the full potential of music therapy in clinical practice.

Nonetheless, it is imperative to recognize the constraints inherent to our investigation. The selection process was confined to the core collection of the Web of Science database, potentially narrowing the scope of our conclusions regarding the field at large. Despite this constraint, it is important to acknowledge the WoSCC database’s reputation as one of the most authoritative sources for bibliometric analyses. Furthermore, the restriction to English-language studies may have excluded relevant research published in other languages, slightly skewing the breadth of our review.

## Data Availability

No datasets were generated or analysed during the current study.
